# Cross-Study Homogeneity of Psoriasis Gene Expression in Skin across a Large Expression Range

**DOI:** 10.1371/journal.pone.0052242

**Published:** 2013-01-04

**Authors:** Jeannette Bigler, Hugh A. Rand, Keith Kerkof, Martin Timour, Christopher B. Russell

**Affiliations:** Medical Sciences, Amgen Inc., Seattle, Washington, United States of America; University Hospital Hamburg-Eppendorf, Germany

## Abstract

**Background:**

In psoriasis, only limited overlap between sets of genes identified as differentially expressed (psoriatic lesional vs. psoriatic non-lesional) was found using statistical and fold-change cut-offs. To provide a framework for utilizing prior psoriasis data sets we sought to understand the consistency of those sets.

**Methodology/Principal Findings:**

Microarray expression profiling and qRT-PCR were used to characterize gene expression in PP and PN skin from psoriasis patients. cDNA (three new data sets) and cRNA hybridization (four existing data sets) data were compared using a common analysis pipeline. Agreement between data sets was assessed using varying qualitative and quantitative cut-offs to generate a DEG list in a source data set and then using other data sets to validate the list. Concordance increased from 67% across all probe sets to over 99% across more than 10,000 probe sets when statistical filters were employed. The fold-change behavior of individual genes tended to be consistent across the multiple data sets. We found that genes with <2-fold change values were quantitatively reproducible between pairs of data-sets. In a subset of transcripts with a role in inflammation changes detected by microarray were confirmed by qRT-PCR with high concordance. For transcripts with both PN and PP levels within the microarray dynamic range, microarray and qRT-PCR were quantitatively reproducible, including minimal fold-changes in IL13, TNFSF11, and TNFRSF11B and genes with >10-fold changes in either direction such as CHRM3, IL12B and IFNG.

**Conclusions/Significance:**

Gene expression changes in psoriatic lesions were consistent across different studies, despite differences in patient selection, sample handling, and microarray platforms but between-study comparisons showed stronger agreement within than between platforms. We could use cut-offs as low as log10(ratio) = 0.1 (fold-change = 1.26), generating larger gene lists that validate on independent data sets. The reproducibility of PP signatures across data sets suggests that different sample sets can be productively compared.

## Introduction

Psoriasis is a common chronic inflammatory skin disease characterized by keratinocyte hyperproliferation, changes in epidermal differentiation, and immune infiltrates in lesions. Histological analysis reveals patterns of abnormal epidermal hyperplasia and differentiation [1]. Changes in the epidermis appear to be preceded by immune activation as evidenced by increased numbers of T-lymphocytes and dendritic cells [2]. These cellular changes in psoriatic lesions are reflected in altered gene expression profiles. A number of RNA expression profiling studies have identified many genes that are highly regulated in PP (psoriatic lesional) versus PN (psoriatic uninvolved) skin, drawing different, but not necessarily conflicting, conclusions about several aspects of the disease [3], [4], [5], [6], [7].

A common approach to comparing PP versus PN differences between data sets is to generate lists of differentially expressed genes (DEG) using thresholds of magnitude of difference and statistical significance to classify genes as differentially expressed. This strategy was used in several studies exploring differences between PP and PN skin in psoriasis [3], [4], [5], [6]. In these publications, a measure of significance (false-discovery rate or q-value) was combined with a 2-fold change cut-off to identify sequences that were differentially expressed. This resulted in DEGs that represented between 2% and 7% of all the probe sets on the array. One recent publication examined how well the results from the published findings align [8]. One conclusion from this study was that simply comparing published DEG lists failed to generate consensus between sample sets. Gene Set Enrichment Analysis (GSEA) [9] was then used to compare lists of genes with significantly altered expression in psoriatic lesions as measured in different data sets using Affymetrix arrays. This did identify common pathways, but the gene set overlap was limited to several hundred probe sets – a number that we shall show under-represents the number of consistently differentially expressed probe sets. While lists of DEGs can be useful for directing future experiments and can be used for biological interpretation, they are not necessarily a good starting point for assessing the overall agreement between different data sets focused on the same biological problem [10]. If used for this purpose, some modification to DEG generation is necessary to prevent the false impression of poor agreement between data sets [11], [12]. Sets of DEGs produced by p-value cut-offs are known to be unstable even to relatively minor experimental differences [11], [12]; this problem is compounded by differences in sample set characteristics such as patient populations, sample preparation, and analysis methodologies.

Here we expanded the exploration of gene expression in psoriasis, and compared the data sets to each other using a global approach. We also addressed the question of whether relatively small changes between PP and PN skin (less than 2-fold at a given p-value often used as a minimum threshold to identify differentially expressed genes) are reproducible across different psoriasis sample sets. Of particular interest are cytokines involved in Th1 and Th17 biology (IL-12, IFNg, IL-23, IL-17A and IL-17F), which have been shown to play a role in psoriasis [5]. Signals for these transcripts have been difficult to detect in the previously published data sets. All the published data on gene expression profiling in psoriasis have been generated with cRNA target and we have routinely used cDNA targets for microarray hybridization. RNA-DNA hybrids and DNA-DNA hybrids have different propensities for cross-hybridization [13] and it is conceivable that a transcript is differentially detectable between the two target types. Therefore we also examined potential advantages of using cDNA target instead of cRNA in detecting signals for Th1 and Th17 genes. For genes of the IL-17 pathway as well as additional transcripts with a role in inflammation, we also explored the extent to which PP/PN expression differences could be replicated by qRT-PCR.

## Results

In this study, we used eight microarray sample sets to explore commonalities and differences in the psoriasis lesions (Table 1). Of these eight sample sets, all generated on related versions of Affymetrix microarrays, three were generated by our group and five were previously published microarray sample sets. Two of the sample sets from our group were collected either as part of clinical trials where we had access to samples or through procurement from Asterand. The third set was generated from RNAs from GSE11903 that were generously provided to us by Dr. James Krueger. All the studies from which data were compared in this study included only plaque psoriasis that ranged from mild to severe. The original Gudjonsson set (GSE13355) was split into two sets due to a batch effect apparent as different mean intensities between the batches (data not shown). Six of the data sets were generated on HG-U133_Plus_2 arrays with 54,613 probe sets, and two were generated on HG-U133A or HGU133A_2 arrays with 22,215 probe sets representing a subset of the HG-U133_Plus_2 probe set content. These data sets comprise the majority of published large-scale microarray analyses on psoriasis gene expression and are based on various versions of Affymetrix microarrays with identical probe sequences. Thus, results for given probe sets should be relatively comparable. One published data set was not included in this analysis [7] because the samples were analyzed on HG-U95 arrays, which is an older microarray chip and contains only approximately 12,000 probe sets. Several transcripts of interest e.g. IL-17F, IL-1F6, IL-1F9, and FOXP3, are not included on this array. Given the number of data sets compared in this analysis, we did not expect exclusion of this data set to have a major impact on the conclusion. For these comparisons we describe the sample sets in terms of: (1) microarray hybridization using labeled cRNA, (2) microarray hybridization using labeled cDNA, and (3) qRT-PCR.

**Table 1:** Psoriasis Data Sets.

Study	GEO Accession	Tissue Type*	# Samples in Study	# Samples in Analysis	Array Type	Labeling Method	Reference
Yao	GSE14905	LS	32	25	U133_Plus_2	cRNA	Yao et al. 2008
		NL	28	25			
Reischl	GSE6710	LS	13	13	U133A	cRNA	Reischl et al. 2007
		NL	13	13			
Zaba (GSE11903)	GSE11903	LS	15	14	U133A_2	cRNA	Zaba et al. 2009
		NL	15	14			
		Week12 LS	15	8			
Gudjonsson	GSE13355	LS	39	37	U133_Plus_2	cRNA	Gudjonsson et al. 2010
Low		NL	39	37			
Gudjonsson		LS	19	19			
High		NL	19	19			
Zaba (Amgen)	GSE41664	LS	15	15	U133_Plus_2	cDNA	in-house
		NL	15	15			
		Week12 LS	9	8			
Asterand	GSE41664	LS	14	14	U133_Plus_2	cDNA	in-house
		NL	14	14			
NCT00867100	GSE41664	LS	24	24	U133_Plus_2	cDNA	in-house
		NL	24	24			

* LS = lesional, NL = non-lesional.

For our microarray analyses, we used a sample preparation method for microarrays that generates labeled cDNA instead of cRNA [13]. Using cDNA target has been shown to result in reduced cross-hybridization compared to cRNA target [13] and has the potential to create a larger set of probe sets and transcripts that can be reliably detected. With this method, transcripts for IFNG, the IL-23 subunits (IL23A, IL12B), and the Th17 cytokine IL-17F were detected as differentially expressed, whereas in the published studies using labeled cRNA on Affymetrix chips with the same probe sets, the transcripts for these genes were not classified as differentially expressed between PP and PN skin samples under the cut-offs used in those publications (Table 2) [3], [4], [5], [6]. With the low background cDNA hybridization, higher fold-changes were detected for many of the genes with low PN expression, including IDO1, IFNG, IL12B, IL17F, and VNN3. While differential expression was higher with cDNA target for transcripts with low expression, the fold-change for transcripts with higher levels of expression such as CD3G, CCL5, and IL1F9 tended to be higher with cRNA. The fold-changes for the genes shown in Table 2 were not equally detected across the cRNA data sets. Amongst the cRNA data sets higher fold-changes were estimated from the Gudjonsson High set. This set had a higher average intensity than the Gudjonsson Low or Yao sets, indicating that signal intensity as well as hybridization background may play a role.

**Table 2:** Fold-Changes Derived from Comparing PP and PN Skin Samples for Selected Probe Sets Across Data Sets and Technologies.

		qRT-PCR ratio (95% CI)#	Microarray fold-change (95% CI)#					
Gene Symbol	dCT*	Asterand	Asterand	NCT 00867100	Zaba (Amgen)	Gudjonsson High	Gudjonsson Low	Yao
IL17A	−17.4	35.6 (11.2,61.8)	9.3 (5.8,24.5)	7.2 (5.0,15.1)	8.3 (6.3,14.5)	9.1 (6.2,20.0)	4.8 (3.7,7.9)	5.4 (3.6,11.7)
IL17F	−17.4	16.8 (5.4,25.7)	3.0 (2.1,6.3)	2.6 (2.0,4.6)	3.7 (2.6,7.8)	1.9 (1.5,2.9)	1.4 (1.2,1.9)	1.8 (1.4,2.7)
CXCL6	−17.2	4.9 (1.7,7.6)	3.9 (1.9,15.5)	6.0 (2.6,31.6)	11.7 (5.4,56.2)	4.7 (3.1,10.7)	2.7 (2.1,4.5)	2.3 (1.8,3.9)
IL20	−17.1	72.9 (15.2,121)	6.9 (3.9,21.9)	8.3 (6.3,14.5)	8.1 (5.9,15.5)	5.1 (3.8,9.3)	5.5 (4.0,10.5)	4.9 (2.8,14.8)
IL19	−16.9	153 (27.0,263)	10.0 (5.8,30.2)	21.9 (14.1,52.5)	14.1 (7.8,46.8)	33.1 (16.6,131)	18.6 (10.5,58.9)	10.2 (5.6,33.9)
IL1F6	−16.9	549 (152,902)	14.8 (8.1,49.0)	7.9 (4.7,22.9)	14.5 (10.7,26.3)	9.8 (6.2,24.5)	5.9 (4.5,10.2)	5.2 (3.6,11.0)
IL22	−16.8	7.4 (3.1,10.9)	12.3 (5.1,70.8)	7.9 (4.9,20.9)	11.0 (5.8,39.8)	2.2 (1.5,4.6)	2.2 (1.9,3.1)	3.2 (2.2,7.1)
IL12B	−16.8	18.6 (5.4,27.2)	14.5 (7.4,55.0)	5.1 (3.6,10.2)	10.7 (6.6,28.2)	2.7 (2.1,4.5)	1.7 (1.5,2.1)	2.1 (1.8,2.9)
IL13	−16.7	1.7 (0.7,2.5)	1.0 (1.0,1.3)	1.0 (0.9,1.3)	1.0 (0.9,1.2)	1.1 (0.9,1.5)	1.0 (0.9,1.4)	1.2 (1.1,1.5)
IL26	−16.7	14.5 (6.1,21.8)	14.8 (9.8,33.9)	7.1 (5.6,11.2)	10.0 (7.4,18.2)	4.3 (3.5,6.2)	2.5 (2.2,3.3)	2.8 (2.4,3.9)
IL23A	−16.4	11.7 (4.3,18.1)	2.5 (1.7,4.9)	2.0 (1.6,3.0)	3.3 (2.6,5.5)	1.9 (1.6,2.6)	1.4 (1.2,1.7)	1.5 (1.3,1.9)
CAMP	−16.4	4.3 (1.6,6.5)	1.6 (1.3,2.7)	3.4 (2.7,5.4)	2.2 (1.7,3.7)	2.0 (1.6,3.0)	2.3 (1.9,3.4)	2.3 (1.9,3.7)
IFNG	−16.2	10.8 (3.9,16.9)	6.3 (4.8,11.0)	6.8 (4.6,14.8)	5.8 (4.2,11.0)	1.9 (1.7,2.5)	1.5 (1.4,1.9)	2.3 (1.9,3.7)
IDO1	−15.6	11.8 (4.8,15.6)	12.0 (6.6,39.8)	14.1 (8.5,38.9)	10.7 (7.1,24.5)	7.6 (5.0,17.4)	5.4 (4.3,8.5)	4.4 (3.1,8.7)
VNN3	−15.4	84.3 (14.5,107.1)	43.7 (25.7,125)	85.1 (64.6,147)	16.2 (11.2,33.9)	20.9 (14.5,43.7)	8.9 (7.1,14.1)	14.8 (11.0,26.9)
ICOS	−14.6	8.2 (4.0,12.1)	4.0 (3.1,6.6)	3.7 (3.0,5.6)	3.7 (3.0,5.9)	3.6 (2.8,6.0)	2.6 (2.2,3.5)	2.8 (2.0,5.4)
CCL8	−12.4	1.6 (0.5,2.5)	1.4 (1.2,2.2)	2.0 (1.6,3.2)	2.5 (2.0,3.7)	2.1 (1.8,3.1)	2.5 (2.1,3.2)	3.2 (2.4,5.9)
IL7R	−11.3	5.2 (2.6,7.6)	5.8 (3.0,21.9)	4.6 (3.3,8.7)	8.7 (4.3,36.3)	3.3 (2.5,6.0)	3.2 (2.8,4.5)	7.2 (4.8,16.6)
CHRM3	−10.9	0.3 (0.1,0.5)	0.4 (0.3,0.6)	0.3 (0.2,0.5)	0.4 (0.4,0.6)	0.7 (0.5,1.0)	0.5 (0.5,0.7)	0.4 (0.2,0.7)
FOXP3	−10.7	4.1 (2.1,5.8)	1.0 (1.0,1.3)	1.0 (0.9,1.2)	1.0 (0.9,1.3)	0.9 (0.8,1.3)	0.8 (0.7,1.1)	0.9 (0.8,1.2)
CD3G	−10.3	2.6 (1.4,3.7)	2.0 (1.6,3.2)	1.8 (1.5,2.3)	2.0 (1.5,3.7)	2.2 (1.5,4.6)	2.9 (2.0,5.8)	3.0 (2.1,6.3)
TNF	−10.2	1.5 (0.9,2.0)	1.6 (1.2,2.6)	1.1 (0.9,1.6)	1.4 (1.2,1.7)	1.4 (1.3,1.8)	1.5 (1.4,1.8)	1.7 (1.5,2.4)
TNFRSF11B	−9.8	1.0 (0.6,1.4)	1.3 (1.1,1.7)	1.3 (1.1,1.6)	1.3 (1.2,1.7)	1.4 (1.2,1.9)	1.3 (1.1,1.6)	1.1 (0.9,1.7)
TNFSF11	−9.2	1.3 (1.1,1.5)	1.4 (1.1,2.6)	1.1 (0.9,2.0)	1.0 (0.7,1.8)	1.7 (1.4,2.4)	1.1 (1.0,1.5)	1.3 (1.2,1.7)
CCL5	−9.0	1.4 (0.9,1.9)	1.8 (1.4,3.0)	1.6 (1.3,2.3)	1.8 (1.5,2.8)	3.0 (2.3,5.2)	2.6 (2.0,4.4)	4.9 (3.4,10.2)
TNFRSF11A	−9.0	0.9 (0.8,1.1)	1.5 (1.3,1.9)	1.5 (1.3,1.9)	1.6 (1.4,2.1)	1.2 (1.1,1.4)	1.3 (1.2,1.6)	1.6 (1.4,1.9)
CCL22	−8.3	4.2 (2,6.2)	3.9 (2.9,7.1)	3.3 (2.5,6.0)	2.6 (2.0,4.6)	2.4 (1.9,3.6)	5.8 (4.7,8.7)	9.5 (6.5,20.9)
S100A8	−7.4	585 (131,908)	6.0 (4.5,11.0)	4.7 (3.4,8.9)	2.0 (1.7,3.1)	5.0 (4.0,7.9)	8.9 (7.6,12.3)	7.4 (6.2,10.7)
IL1F9	−6.9	25.6 (8.3,42.7)	8.7 (6.9,13.8)	13.2 (11.0,19.1)	5.4 (4.6,7.4)	15.5 (12.9,22.4)	17.8 (14.1,28.2)	12.6 (10.7,17.4)
CRAT	−6.2	0.4 (0.1,0.6)	0.3 (0.2,0.7)	0.3 (0.2,0.5)	0.3 (0.2,0.5)	0.5 (0.4,1.0)	0.4 (0.3,0.8)	0.5 (0.3,0.9)
KRT16	−6.0	55.2 (13.2,100)	5.8 (4.7,8.7)	6.3 (5.6,7.9)	4.8 (3.5,9.1)	5.5 (4.6,7.9)	17 (12.3,32.4)	12.3 (9.5,20.4)

* Expression levels in PN tissue expressed as ΔCt of average of housekeeping genes and average of transcript of interest.

# CI = confidence interval.

### Differential Expression in Psoriatic Lesions Across Various Data Sets

In previous publications, the number of differentially expressed probe sets or transcripts reported for comparisons between PP and PN samples was less than 1000 for sample sizes of up to about 60 PP/PN pairs [8]. All of these analyses classified probe sets as differentially expressed using a combination of two different threshold filters, a quantitative filter represented by a minimum fold-change and a threshold for statistical significance represented by either an unadjusted p-value or a false discovery rate. Applying the same data processing method for both internal and available external samples sets (Table 3), in all the data sets run on HG-U133_Plus_2 arrays, we found >16,000 probe sets differentially expressed in PP versus PN tissues at a p-value of ≤0.05 and in the absence of a fold-change threshold (Table 3). Applying the commonly used 2-fold change cut-off to our three data sets reduced the number of probe sets categorized as differentially expressed to <3800.

**Table 3:** Probe Sets Differentially Expressed at p-value Cut-offs or p-value and Fold-Change Cut-offs.

A Data Sets on U133_Plus_2													
	Sample Set	Differentially Expressed Sequences^a^											
		p≤0.05						p≤0.05 and Fold-change≥2					
		Up		Down		Total		Up		Down		Total	
	Asterand	8,128	(0.148)	8,161	(0.149)	16,289	(0.298)	1,453	(0.027)	1,651	(0.030)	3,104	(0.057)
	NCT00867100	9,565	(0.175)	11,066	(0.202)	20,631	(0.377)	1,862	(0.034)	1,952	(0.036)	3,814	(0.070)
	Zaba (Amgen)^b^	8,296	(0.152)	9,136	(0.167)	17,432	(0.319)	1,556	(0.028)	1,478	(0.027)	3,034	(0.056)
	Gudjonsson High	9,264	(0.169)	10,660	(0.195)	19,924	(0.364)	1,363	(0.025)	786	(0.014)	2,149	(0.039)
	Gudjonsson Low	9,666	(0.177)	11,624	(0.212)	21,290	(0.389)	1,290	(0.024)	678	(0.012)	1,968	(0.036)
	Yao	9,567	(0.175)	11,827	(0.216)	21,394	(0.391)	1,681	(0.031)	1,648	(0.030)	3,329	(0.061)
**B All Data Sets with Content Restricted to U133_A**													
	**Sample Set**	**Differentially Expressed Sequences** a											
		**p≤0.05**						**p≤0.05 and Fold-change≥2**					
		**Up**		**Down**		**Total**		**Up**		**Down**		**Total**	
	Asterand	4,299	(0.194)	3,478	(0.157)	7,777	(0.350)	1,453	(0.065)	1,651	(0.074)	3,104	(0.140)
	NCT00867100	5,247	(0.236)	4,382	(0.197)	9,629	(0.433)	1,862	(0.084)	1,952	(0.088)	3,814	(0.172)
	Zaba (Amgen)	4,328	(0.195)	3,937	(0.177)	8,265	(0.372)	1,556	(0.070)	1,478	(0.067)	3,034	(0.137)
	Zaba (GSE11903)	4,241	(0.191)	4,604	(0.207)	8,845	(0.398)	736	(0.033)	724	(0.033)	1,460	(0.066)
	Gudjonsson High	5,480	(0.247)	4,410	(0.199)	9,890	(0.445)	1,363	(0.061)	786	(0.035)	2,149	(0.097)
	Gudjonsson Low	5,666	(0.255)	5,090	(0.229)	10,756	(0.484)	1,405	(0.063)	783	(0.035)	2,188	(0.098)
	Yao	5,291	(0.238)	5,046	(0.227)	10,337	(0.465)	1,681	(0.076)	1,648	(0.074)	3,329	(0.150)
	Reischl	4,285	(0.193)	4,947	(0.223)	9,232	(0.416)	639	(0.029)	697	(0.031)	1,336	(0.060)

a # probe sets (%).

b Baseline samples only.

**Figure 1:**
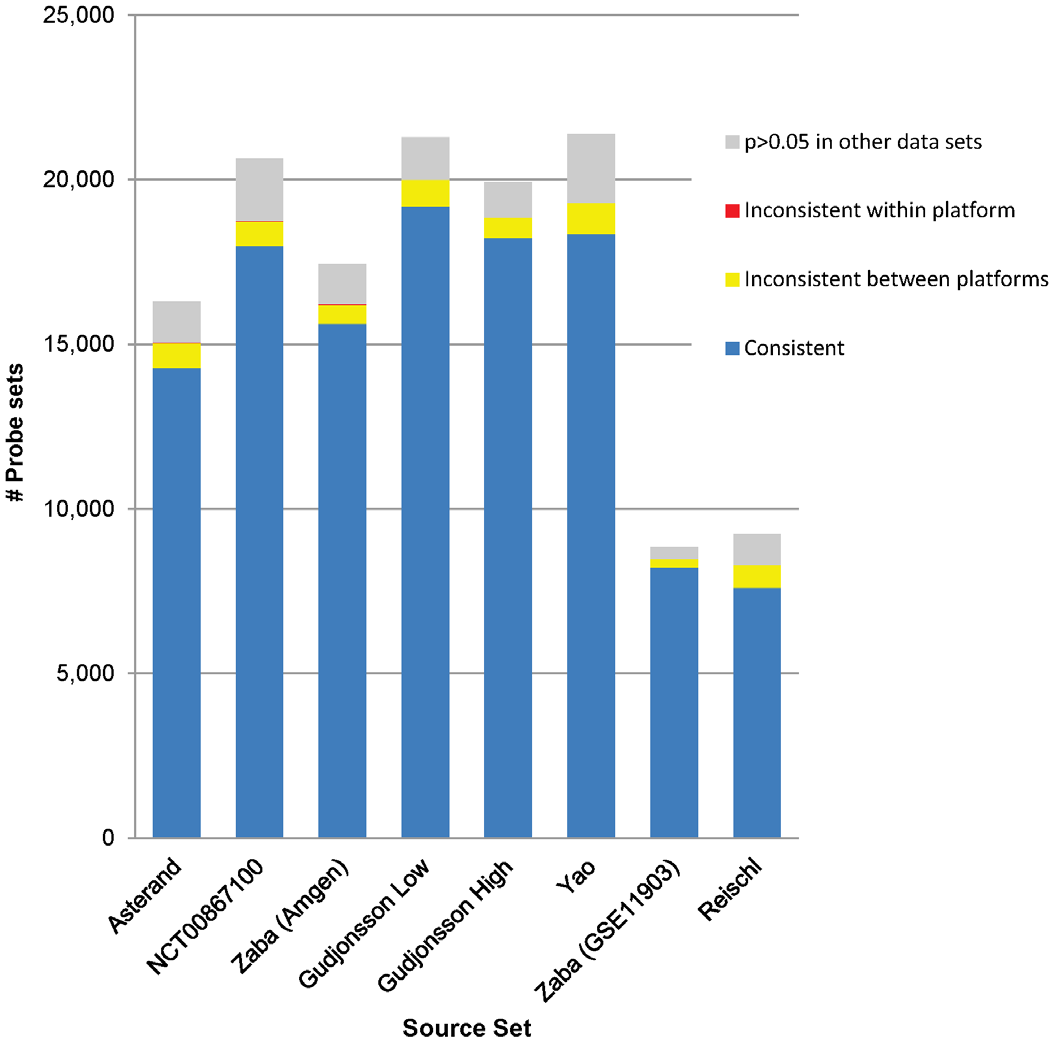
Comparison of Differential Expression Across Data Sets. For each data set a list of probe sets with differential expression at p≤0.05 was generated and compared to all the other data sets. The probe sets were then categorized into four different groups according to the extent of agreement between the source data set and the other data sets: i) “consistent” meant that there was at least one other data set in which the probe set showed differential expression in the same direction with p≤0.05 and no data sets with differential expression in the opposite direction with p≤0.05; ii) “inconsistent between platforms” indicated that there was at least one data set from the other platform with differential expression at p≤0.05 in the opposite direction; iii) the “inconsistent within platform” group contained probe sets with differential expression at p≤0.05 in different directions within the same platform; and iv) the “p>0.05 in all other” group contained probe sets where the source set was the only one with significant differential expression. The number of probe sets with differential expression in the Zaba (GSE11903) and the Reischl sets were smaller because samples were run on U133A arrays, which contain only 22,215 probe sets.

Several groups have characterized the transcriptome in psoriatic lesional skin [3], [4], [5], [6], [7] by comparing gene lists. The results from these analyses can appear discordant [8] since simple comparison of lists of regulated genes is highly sensitive to threshold effects [12], [14]. GSEA has been used to identify similarities across different sample sets [8], but there are other ways to view experiment concordance. One can instead ask: How many genes that are identified as differentially expressed using specific cut-offs in one sample set (the “source” sample set) appear as differentially expressed in the same direction in other sample sets? In Figure 1, we selected one data set as the source set and used a p-value cut-off of ≤0.05 to identify a set of differentially expressed probe sets. We then compared this list of probe sets with the other data sets and categorized the probe sets into four groups. These groups characterize the possible levels of agreement across data sets (see also legend to Figure 1): i) “consistent”; ii) “inconsistent between platforms”; iii) “inconsistent within platform”; and iv) the “p>0.05 in all other data sets”. This comparison showed good agreement between data sets. For all source sets, differential expression for >85% of the probe sets identified as differentially expressed in a source set was also identified in at least one other data set, with no contradictions in any set. For a small number of probe sets (<8%) there were inconsistencies between the cDNA and cRNA platforms and for a very small number (<0.2%) inconsistencies within a platform. Each source set produced a number of probe sets called differentially expressed only in that set. The proportion of these probe sets could represent up to 10% of the differentially expressed probe sets but on average was only 6%.

For this comparison across data sets we used a single p-value cut-off. The robustness of the conclusions from the comparison to the effects of p-value and fold-change cut-offs can be investigated. We used pair-wise comparisons to ask how many genes that are identified as up-regulated using varying cut-offs in one sample set (the “source” sample set) do appear as up-regulated in a second sample set (the “target” sample set). We can then refine this question: How many of the selected genes from the source sample set change in the same or opposite direction in the target sample set when that direction is well determined (p≤0.05) in the target experiment? The target experiment filter in the latter question eliminates probe sets that have larger variances in the target data set, either because of noise in the target data set population or in the target data set technology. We then tested different restrictions on fold-change or p-value to examine the effects on concordance between data sets.

To compare the different data sets, we first normalized the data using the same method for in-house and previously published data sets. Because differential gene expression analysis is commonly performed with data transformed into logarithmic space, the PP/PN fold-changes were expressed as log10(ratio). The log10(ratio) and p-values were estimated using a linear model and t-test for each probe set in each data set separately. We then applied different cut-offs for log10(ratio) and p-value to classify genes as up- or down-regulated. When no cut-off was used, any log10(ratio) >1 was classified as up-regulated and any log10(ratio) <1 was classified as down-regulated. In pair-wise comparisons, each probe set was classified as agreeing if it was up-regulated (or down-regulated) in both sample sets. The number of agreeing and disagreeing probe sets in each pair-wise comparison were counted and plotted using various cut-offs.

For all possible pair-wise comparisons between data sets in this analysis we examined results produced by four different p-value and log10(ratio) cutoffs: i) no log10(ratio) or p-value cut-off, ii) p≤0.05 in the source set only, iii) log10(ratio)≥0.1and p≤0.05 in the source set only and iv) log10(ratio)≥0.1and p≤0.05 in the source set as well as p≤0.05 in the target set. The smaller number of probe sets contained on the HG-U133A and HG-U133A_2 arrays compared to the HG-U133_Plus_2 represents a non-random subset that consists of a large number of well-expressed probe sets. This may bias some of the comparisons. After applying the cut-off values to the source data set, the fraction of selected probe sets agreeing in the target set was calculated (Table 4). The reciprocal pairs have slightly different fractions of probe sets agreeing because the source set changed. Table 4A shows the fraction of agreement between all the data sets using no log10(ratio) or p-value cut-offs. There were more differences across the hybridization platforms than between different data sets within a platform. In the absence of any cut-offs, the apparent agreement ranged from 0.674 to 0.720 for cRNA to cDNA comparisons and from 0.773 to 0.808 for within platform comparisons (Table 4A). Lack of agreement can be due to sample population differences, platform differences, or different patient selection criteria. Perfect reproducibility would generate 100% agreement. The agreement level of >75% within a platform suggested that more than half the total probe sets on the HG-U133_Plus_2 chips were reproducibly modulated between PP and PN skin. The difference when comparing changes between cRNA hybridization experiments and cDNA hybridization experiments suggested that a substantial fraction of probe sets were well measured on each platform.

**Table 4:** Proportion of Probe Sets Agreeing in the Direction of Expression Change (Log10(ratio)) between PN and PP Skin.

A Comparison without cut-offs on p-value or log10(ratio)									
	Source Set	Asterand	NCT 00867100	Zaba (Amgen)	Zaba (GSE11903)	Gudjonsson High	Gudjonsson Low	Yao	Reischl
	**Asterand** *		0.8075	0.7732	0.7213	0.6861	0.6791	0.6741	0.6946
	**NCT00867100** *	0.8075		0.7909	0.7461	0.7199	0.7129	0.7122	0.6990
	**Zaba (Amgen)** *	0.7732	0.7909		0.7595	0.7177	0.7095	0.7087	0.7064
	**Zaba (GSE11903)** #	0.7213	0.7461	0.7595		0.7764	0.7697	0.7708	0.7570
	**Gudjonsson High** #	0.6861	0.7199	0.7177	0.7764		0.8059	0.7769	0.7222
	**Gudjonsson Low** #	0.6791	0.7129	0.7095	0.7697	0.8059		0.7856	0.7210
	**Yao** #	0.6741	0.7122	0.7087	0.7708	0.7769	0.7856		0.7075
	**Reischl** #	0.6946	0.6990	0.7064	0.7570	0.7222	0.7210	0.7075	
**B Comparison using cut-offs of p-value ≤0.05 in source set**									
	**Source Set**	**Asterand**	**NCT 00867100**	**Zaba (Amgen)**	**Zaba (GSE11903)**	**Gudjonsson High**	**Gudjonsson Low**	**Yao**	**Reischl**
	**Asterand** *		0.9850	0.9649	0.9009	0.8768	0.8737	0.8610	0.8603
	**NCT00867100** *	0.9742		0.9646	0.8981	0.8954	0.8868	0.8837	0.8438
	**Zaba (Amgen)** *	0.9629	0.9747		0.9292	0.9067	0.8973	0.8971	0.8761
	**Zaba (GSE11903)** #	0.8911	0.9185	0.9320		0.9502	0.9433	0.9392	0.9296
	**Gudjonsson High** #	0.8478	0.9011	0.8988	0.9468		0.9862	0.9621	0.8725
	**Gudjonsson Low** #	0.8316	0.8830	0.8797	0.9267	0.9847		0.9649	0.8592
	**Yao** #	0.8137	0.8761	0.8708	0.9202	0.9566	0.9646		0.8379
	**Reischl** #	0.8373	0.8476	0.8595	0.9206	0.8844	0.8875	0.8640	
**C Comparison using cut-offs of log10(ratio)≥0.1, p-value ≤0.05 in source set**									
	**Source Set**	**Asterand**	**NCT 00867100**	**Zaba (Amgen)**	**Zaba (GSE11903)**	**Gudjonsson High**	**Gudjonsson Low**	**Yao**	**Reischl**
	**Asterand** *		0.9864	0.9693	0.9098	0.8834	0.8789	0.8668	0.8768
	**NCT00867100** *	0.9786		0.9712	0.9048	0.8979	0.8894	0.8853	0.8588
	**Zaba (Amgen)** *	0.9717	0.9805		0.9340	0.9097	0.9006	0.9009	0.8861
	**Zaba (GSE11903)** #	0.9137	0.9300	0.9388		0.9526	0.9484	0.9462	0.9377
	**Gudjonsson High** #	0.8784	0.9156	0.9154	0.9562		0.9878	0.9700	0.9056
	**Gudjonsson Low** #	0.8703	0.9089	0.9058	0.9514	0.9884		0.9792	0.9055
	**Yao** #	0.8368	0.8888	0.8876	0.9366	0.9641	0.9710		0.8684
	**Reischl** #	0.8758	0.8832	0.8911	0.9378	0.9111	0.9135	0.8922	
**D Comparison with cut-offs of log10(ratio)≥0.1 and p≤0.05 in source set; p-value≤0.05 in target set**									
	**Source Set**	**Asterand**	**NCT 00867100**	**Zaba (Amgen)**	**Zaba (GSE11903)**	**Gudjonsson High**	**Gudjonsson Low**	**Yao**	**Reischl**
	**Asterand** *		0.9993	0.9992	0.9859	0.9852	0.9776	0.9707	0.9731
	**NCT00867100** *	0.9993		0.9993	0.9882	0.9880	0.9834	0.9824	0.9643
	**Zaba (Amgen)** *	0.9992	0.9993		0.9913	0.9907	0.9852	0.9843	0.9765
	**Zaba (GSE11903)** #	0.9873	0.9873	0.9916		0.9957	0.9942	0.9917	0.9920
	**Gudjonsson High** #	0.9861	0.9890	0.9920	0.9962		0.9997	0.9985	0.9824
	**Gudjonsson Low** #	0.9819	0.9849	0.9864	0.9958	0.9997		0.9988	0.9831
	**Yao** #	0.9714	0.9829	0.9845	0.9927	0.9983	0.9987		0.9710
	**Reischl** #	0.9752	0.9729	0.9822	0.9930	0.9813	0.9792	0.9716	

* labeled cDNA target.

# labeled cRNA target.

When a p-value ≤0.05 cut-off in the source set was used, the proportion of agreeing probe sets increased to a range of 0.814 to 0.932 for cross-platform comparisons and from 0.838 to 0.986 for comparisons made among data sets run on the same platform (Table 4B). At these cut-offs, there were generally >15,000 probe sets classified as differentially expressed on the larger HG-U133_Plus_2 array. A higher level of disagreement between platforms than within platforms persisted, emphasizing the difference in measurement characteristics, while showing the reproducibility of each. Adding a log10(ratio) cut-off of ≥0.1 to the p≤0.05 cut-off improved the agreement only marginally in the majority of comparisons (Table 4C). The agreement ranged from 0.837 to 0.939 between platforms and from 0.868 to 0.988 within a platform. With an added p-value≤0.05 cut-off in the target set, the proportion of agreeing probe sets increased to a range of 0.964 to 0.992 for between platform and 0.971 to 0.9997 for within platform comparisons (Table 4D), showing that when the data were trimmed to probe sets well measured in each of two data sets, the agreement was extremely high, with some minor remaining platform discrepancy.

The good agreement between platforms continued into comparisons not just between PN and PP, but also between pre-dose and post-dose samples from etanercept treatment [5]. In this case, the original RNA samples and the probe set design were the same but the labeling methodology and the chip type were different. In Table 5, the level of agreement for different statistical cut-offs for PP biopsies pre- and post-etanercept treatment is shown. This level of agreement was comparable to the one obtained for the base-line PP/PN comparison between the Zaba (Amgen) and the Zaba (GSE11903) data sets (Table 4).

**Table 5:** Proportion of Probe Sets^a^ Agreeing in the Direction of Expression Change (Log10(ratio)) between PN and PP Skin between Pre- and Post-Etanercept PP Skin.

Source Set	Zaba (Amgen)^b^	Zaba (GSE11903)^c^
**Without cuts on p-value or log10(ratio)**		
Zaba (Amgen)*	–	0.7432
Zaba (GSE11903)^#^	0.7432	–
**Cut-offs of log10(ratio)>0.1, p-value ≤0.05 in source set**		
Zaba (Amgen)*	–	0.9245
Zaba (Rockefeller)^#^	0.9325	–
**Cut-offs of log10(ratio)≥0.1 and p≤0.05 in source set; p-value<0.05 in target set**		
Zaba (Amgen)*	–	0.9925
Zaba (GSE11903)^#^	0.9974	–

a Only probe sets shared between array types.

b labeled cDNA target.

c labeled cRNA target.

**Figure 2:**
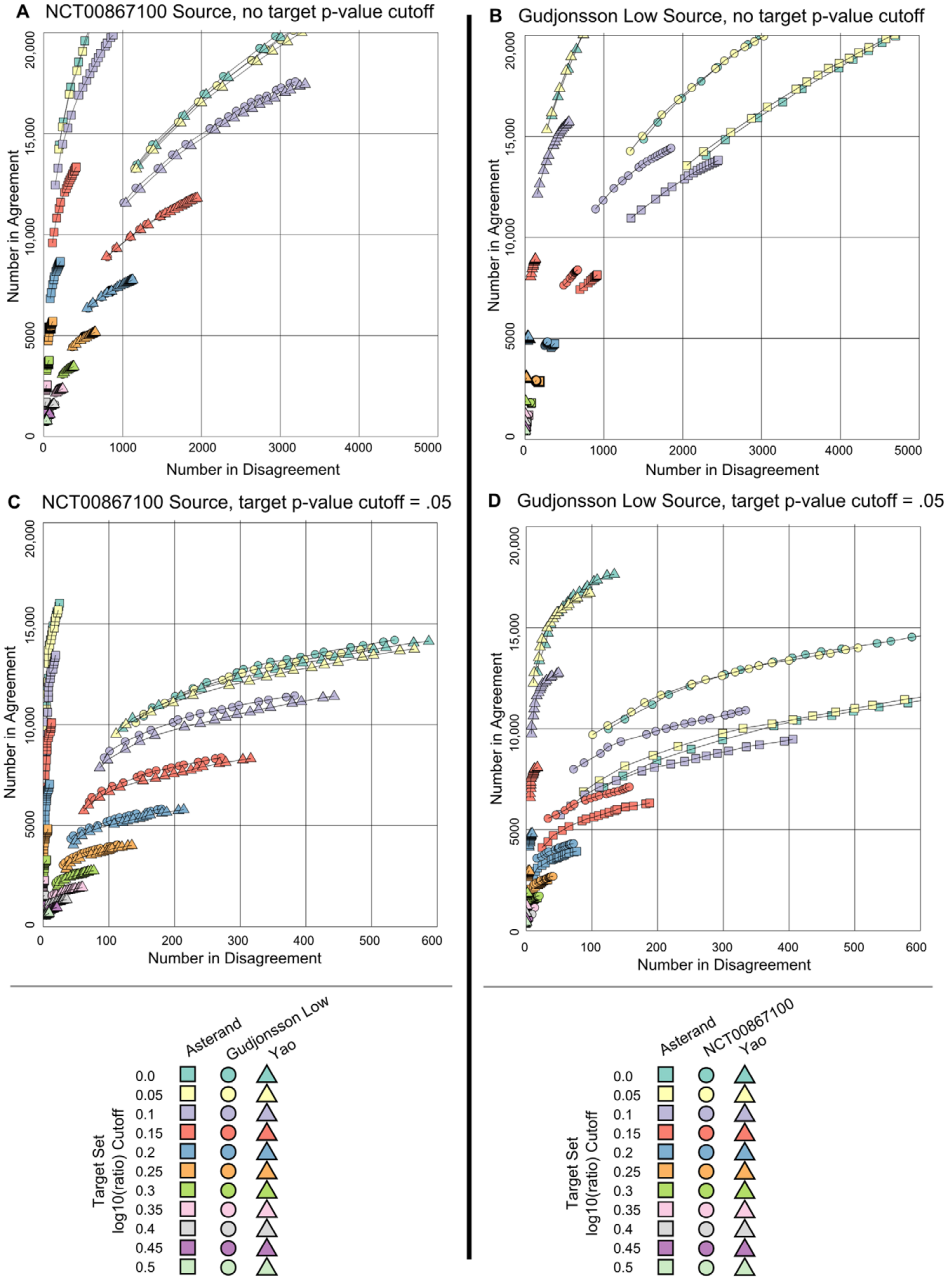
Effect of log10(ratio) and p-value Cut-offs on Overlap of Differentially Expressed Probe Sets. Effects are shown with source sets (A,C) NCT00867100 and (B,D) Gudjonsson low. (A, B): Number of probe sets agreeing and disagreeing for log10(ratios) from 0 to 0.5 in increments of 0.05 and p-values up to 0.2 in increments of 0.01 in the source set without cut-offs in the target set, (C, D): Number of probe sets agreeing and disagreeing from an analysis using log10(ratios) from 0 to 0.5 in increments of 0.5 and p≤0.05 in the source set and p-values up to 0.2 in increments of 0.01 in the target set are shown. Note: X-axis scales in (A, C) differ from those in (B, D).

The agreement between summary results for any pair of data sets depends on the filtering criteria used to produce the summary results. In the case of our data sets (microarray comparisons between two conditions) there are two filtering criteria: a p-value and a fold-change cutoff. Thus, the agreement between any pair as measured by gene-set overlapdepends on four variables. It is challenging to visualize the agreement for multiple pair-wise comparisons as a function of the four filtering variables. We have chosen to plot the number of agreeing and disagreeing probe sets for various pair-wise comparisons of the U133_Plus_2 data sets as a function of the source set cut-offs (Figure 2); we also show this plot for two different target p-value cutoffs. In Figure 2A and 2B, these relationships are shown for log10(ratio) values from 0 to 0.5 in increments of 0.05 and p-values up to 0.2 in increments of 0.01 in the source set without cut-offs in the target set. As the thresholds were relaxed, the number of selected probe sets increased, with some of the selected probe sets in agreement between the two data sets (y-axis value) and some in disagreement (x-axis value). It was apparent that the extent of disagreement was larger for comparisons between two platforms (NCT00867100 vs. Gudjonsson low or Yao) than within a platform (NCT00867100 vs. Asterand). For example, starting with probe sets with a minimal log10(ratio) of 0.1, and a maximal p-value of 0.05 in the source data sets (light purple points), there were approximately 16,000 probe sets in agreement and about 400 or 2.5% in disagreement for data sets from the same platform versus about 14,500 probe sets in agreement and about 1,900 or 13.1% in disagreement for data sets from different platforms. Higher proportions of disagreeing probe sets between platforms were seen consistently across all thresholds tested. This suggested that many of the disagreements may be due to platform-specific differences in the ability to measure certain probe sets.

Clearly, there is a higher level of disagreement between than within platforms. In order to explore how additional threshold criteria on the target data set would affect the number of agreeing and disagreeing probe sets. Figures 2C and 2D show the extent of agreement with a log10(ratio) and p-value cut-off in the source set and additionally a p-value cut-off in the target set (note that the X-axis scale is different from Figures 2A and 2B). When the platform was the same, the level of agreement was extraordinarily high. With low thresholds on the source data set and only a moderate requirement of p≤0.05 on the target data set, there could be in excess of 15,000 agreeing probe sets, with fewer than 20 disagreements.

The absolute number of probe sets where the fold-change disagreed in sign between the source and target sets decreased considerably in Figures 2C and 2D compared to Figure 2A and 2B. At a p-value of 0.05 in source and target set and a log10(ratio) of 0.1 in the source set (Figures 2C and 2D), there were approximately 9,500 probe sets that agreed between platforms and around 170 that disagreed, for a total of less than 2% of probe sets with a discrepant fold-change call. It was evident from these analyses that there was a high level of agreement between data sets, even at log10(ratio) values as low as 0.1 or lower. This high level of agreement was not highly changed throughout the range of target set p-values tested. At the highest source set p-value (0.20) and the lowest log10(ratio) tested, the probe sets disagreeing between sample sets still constituted only about 4% of the probe sets classified as differentially expressed.

**Figure 3:**
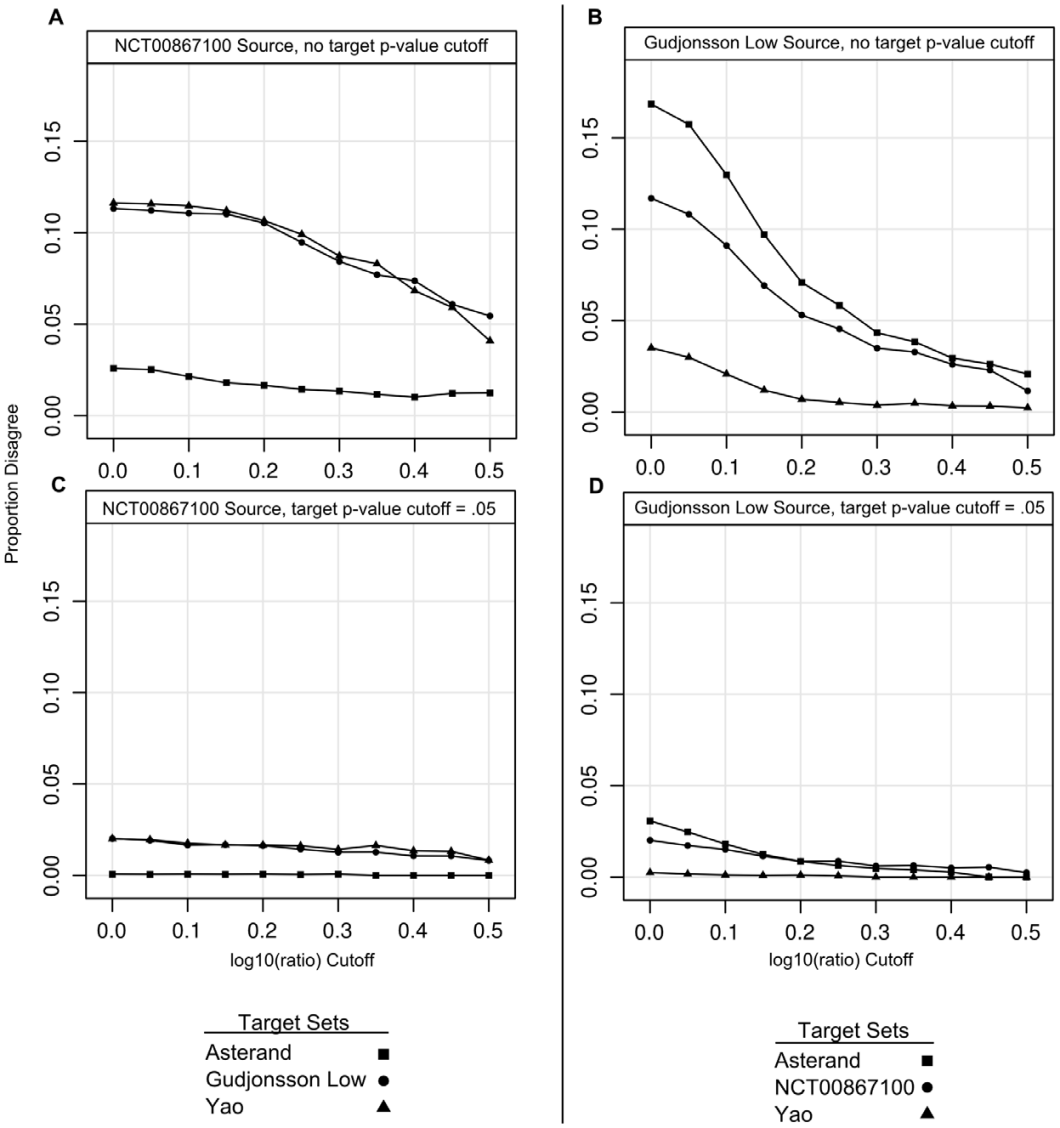
Effect of log10(ratio) on Proportion of “Disagreeing” Probe Sets at a p-value of 0.05 in the Source Set. The data sets are the same as for the data shown in Figure 1. Either NCT00867100 (A, C) or Gudjonsson Low (B, D) were chosen as the source set. The proportion of probe sets disagreeing (out of all the probe sets) is shown for different log10(ratio) cutoffs. A and B: p-value of 0.05 in the source set and no cut-offs in the target sets; C and D: p-value cut-off of 0.05 in source and target set.

For another characterization of the trade-offs made by different fold-change cut-offs, we can select a fixed p-value in the source sets in Figure 2 and then plot the proportion of disagreeing probe sets for different log10(ratio) values (Figure 3). If the fold-change was not required to be well measured in the target set (no p-value cut-off; Figure 3A, B), a relatively high log10(ratio) cut-off of 0.3 to 0.5 was necessary to keep the proportion of disagreeing probe sets around 0.05 when comparing between platforms. In within-platform comparisons, an increase in disagreeing probe sets was observed but it never reached 0.05, even at low log10(ratio) values. If the set of sequences was additionally restricted to those whose fold-change was well measured in the target set (p≤0.05; Figure 3C, D) the platform difference was still detectable. The proportion of disagreeing probe sets increased with decreasing log10(ratio) values but never reached as high as 0.05.

**Figure 4:**
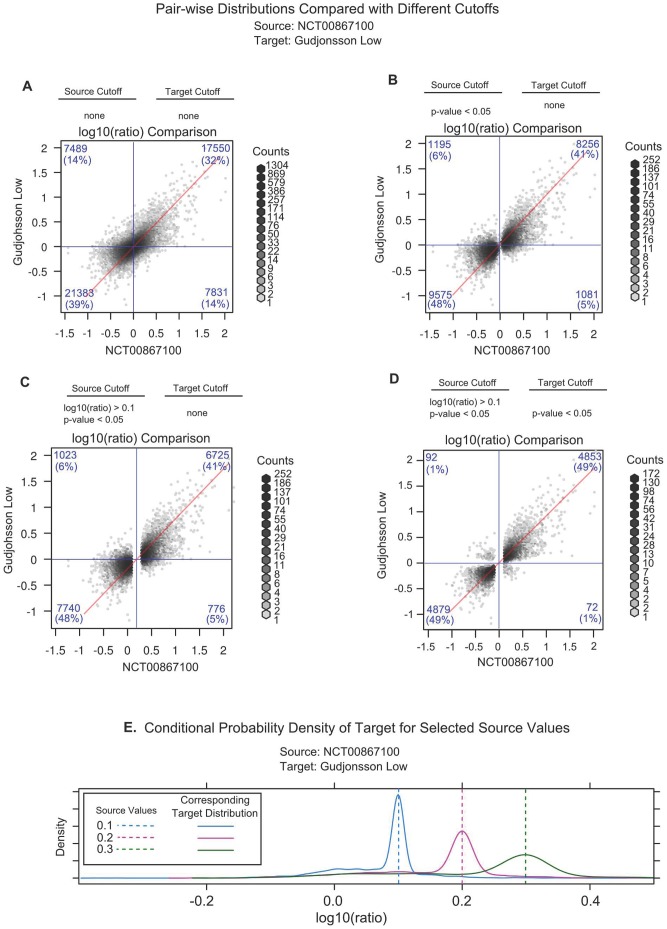
Effect of Implementing Fold-Change and p-value Cut-offs on a Comparison Between Two Experiments. Panels A-D show a hexbin plot comparison of the average log10(ratio) values between the Gudjonsson Low and the NCT00867100 data sets with (A) no cut-offs, (B) a p-value≤0.05 cut-off in the source set only, (C) p-value≤0.05 and log10(ratio)≥0.1 cut-offs only in the source set, and (D) p-value≤0.05 and log10(ratio)≥0.1 cut-offs in the source set and a p-value≤0.05 cut-off in the target set. The numbers in the panel corners indicate the number of data points in those quadrants. Panel E shows average log10(ratio) distributions in the Gudjonsson Low data set (target set) for sequences with log10(ratio) values of 0.100±0.005 (blue), 0.200±0.005 (pink), and 0.300±0.005 (green) in the source set (NCT00867100).

The agreement between probe sets from two data sets from different platforms is shown in Figure 4. High log10(ratios) from one data set were generally identified as having high log10(ratios) in the other. When the set of probe sets was reduced using various thresholds on one (Figure 4B, C) or both (Fig. 4D) of the data sets by application of various thresholds, the correlation increased ((A) 0.65, (B) 0.76, (C) 0.77, (D) 0.88). This increase was primarily due to the p-value restriction. This suggests that comparatively little of the difference between pairs of data sets is due to consistent, well-measured differences, either between the platforms or between the population samples.

The extent of quantitative agreement at low log10(ratio) values can be seen by selecting at set of probe sets within a tight range of log10(ratio) values (±0.005 of a chosen log10(ratio) value) in one data set and looking at the distribution of values for those probe sets in the other data set. While the distribution broadens with increasing log10(ratios), probe sets with specific log10(ratios) in the source data set were distributed in a range around that value in the target data set (Figure 4E). This implies that use of any fold-change cut-off of log10(ratio)>0.1 removes probe sets that perform consistently across experiments. The majority of probe set signals have a quantitatively consistent behavior in psoriasis data sets.

### Differential Expression Determined by Microarray and qRT-PCR

Microarrays have a relatively small assay range, with data compression at high expression levels and background signal and potential for cross-hybridization at low expression levels. This can limit reliable detection and quantitation of differential expression [15]. To evaluate differential expression of a subset of transcripts using an independent method, we used quantitative Reverse Transcription-PCR (qRT-PCR). qRT-PCR assays have high specificity and detection sensitivity [16] and for the purpose of this study qRT-PCR was used as the gold standard. The transcripts were selected from various inflammatory pathways, some of which were considered relevant in psoriasis. Further criteria for selecting probe sets included a range of expression level (both high and low) and under- or over-expression in psoriatic lesions. The transcripts and assays are listed in Table S1. A set of eight PP/PN sample pairs was selected from the Asterand set for qRT-PCR with the intent of representing the largest range of differential expression possible for most of the selected transcripts.

**Figure 5:**
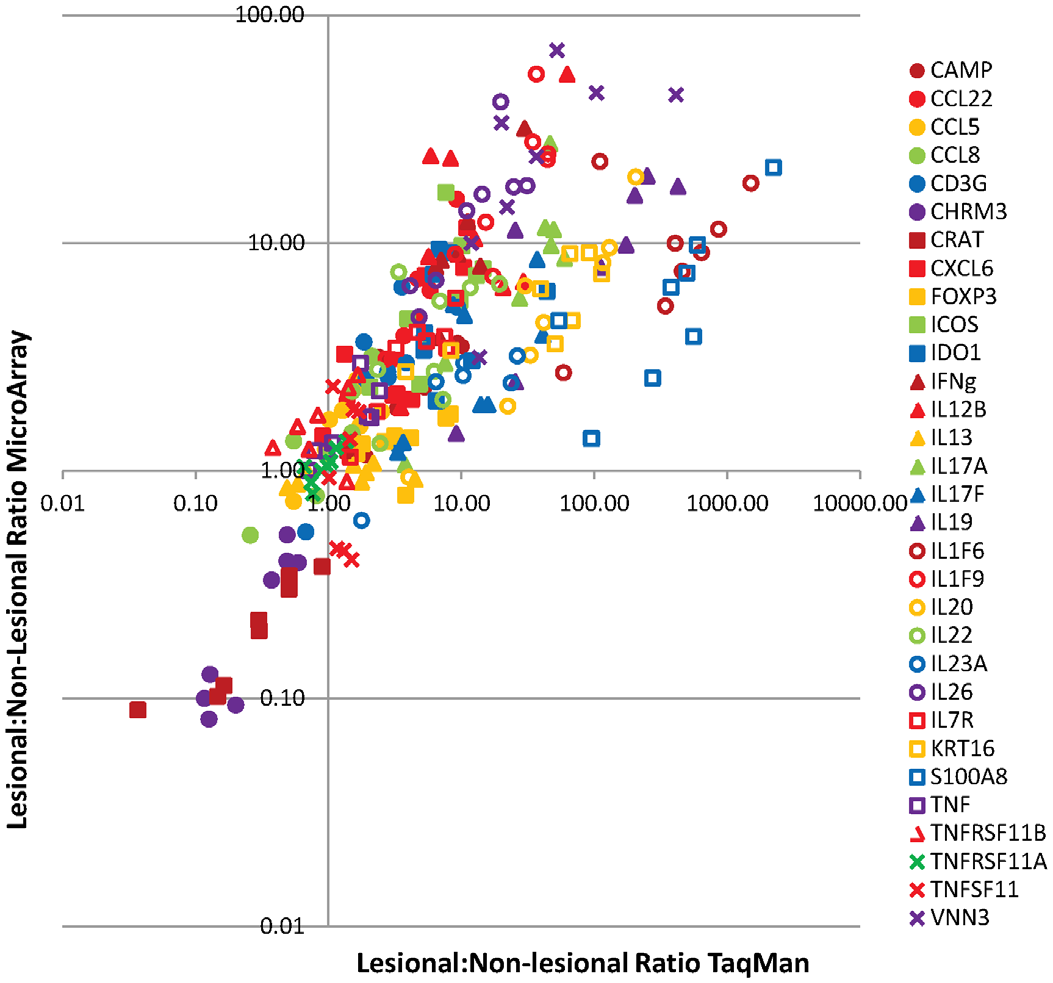
Comparison of Fold-changes in Psoriasis PP/PN Pairs by Microarray and qRT-PCR. Fold-changes for a selection of mostly immune system transcripts were assessed by qRT-PCR and microarray in a subset of eight psoriasis PP/PN skin biopsies from the Asterand set. Transcripts were selected based on relevance to psoriasis, range of expression level and range of fold-changes; patient biopsies were selected based on microarray data so that the range of differential expression was large. The black line indicates complete concordance.

Figure 5 shows the PP/PN ratios for selected analytes on each of the 8 selected sample pairs. For the selected transcripts, most genes had similar fold-change differences across platforms, whether increased or decreased in the PP skin. However, the fold-changes measured by qRT-PCR were clearly larger than those measured by microarray for KRT16 and S100A8, which had relatively high expression levels in PP skin (KRT16: Ct 21 and S100A8: Ct 19 in PP). Microarray did also underreport fold-change for genes with very low baseline expression (Ct>38 in PN skin), most strikingly for IL1F6 (Ct≈28 in PP skin), but also for other cytokines including IL19, IL20, IL17A, and IL17F (all Ct 31 to 34 in PP skin), though the effect was not so large. Only in the case of FOXP3 (Ct 27 to 32) was the apparent background hybridization effect sufficient to eliminate the ability to consistently detect differential expression in the cDNA based microarrays (Ct29 and 31 in PP and PN tissues, respectively). For several other transcripts with low expression, including CAMP, ICOS, IDO1, IFNG, IL12B, and IL22, the qRT-PCR and microarray showed similar average fold-changes. For CHRM3 and CRAT, two genes with decreased expression levels in PP skin, cDNA and cRNA microarray platforms There was also qualitative agreement in a set of genes with decreased expression in PP skin (CHRM3, CRAT) and a control set of intracellular signaling genes with little or no change between PP and PN skin (IL13, TNFSF11, TNFRSF11B). TNFSF11, which was reported to be more highly expressed in one psoriasis lesion [17], was not significantly differentially expressed on the transcript level in our sample sets or in the published data. It should be noted that this correlation was between data from matched individual sample pairs and showed the quantitative agreement between single microarray and qRT-PCR measurements.

## Discussion

Several studies have used whole-genome microarrays or qRT-PCR to explore gene expression changes in psoriatic PP skin. Each study identified biologically relevant sets of genes that were differentially expressed between PP and PN psoriatic skin. Differences between studies can be due to threshold effects, technology choices, or population differences, and do not necessarily reflect pertinent biological differences. We investigated the differences between several microarray data sets and, for a few genes, the differences between qRT-PCR and microarray within one sample set and came to the conclusion that most published data sets are both qualitatively and quantitatively similar.

For genes with expression within the dynamic range of microarray, fold-changes qualitatively agreed between qRT-PCR and microarray analyses with different labeling methodologies for most of the tested genes. Highly expressed genes such as KRT16, IL1F9, and S100A8 suffered fold-change compression on microarray compared to qRT-PCR but remained correlated and were easily classified as differentially expressed. While differential expression was obtained with microarray and qRT-PCR, the latter likely provides a more realistic estimate of the magnitude of differential expression. The hybridization methodology - cRNA vs. cDNA - can also have an effect on ratios. Several cytokine genes with overall low expression but high differential effect in psoriasis (IFNG, IL12B, IL17F, and IL22) displayed higher magnitude changes with cDNA than with cRNA hybridization, whereas the highly expressed gene IL1F9 showed a higher magnitude change with cRNA hybridization. The higher log10(ratio) for low-abundance transcripts with cDNA hybridization applied to several cytokines that are of interest in psoriasis but is not a universal effect of this platform. Other cytokines and receptors with low expression levels and consistently minimal or no changes in psoriasis (IL13, TNFRSF11A, TNFRSF11B, and TNFSF11) showed similar results across all three platforms. While on a population basis there are remarkably consistent quantitative gene expression changes, this does not rule out considerable variation in the fold-changes for particular genes on the individual patient level.

For comparisons across sample sets, microarray results are often summarized by creating a list of genes identified as differentially expressed between two conditions. These sets are usually determined by applying a selection criterion such as a p-value or a fold-change, or often both [3], [4], [5], [6]. This is a somewhat hybrid process in that the results from hypothesis testing (p-value cut-off) are then filtered by a fold-change. As with any such statistical test, failure to reject the null hypothesis does not mean the null hypothesis is true, rather that it is insufficiently supported at the chosen stringency. The fold-change threshold may have been imposed to avoid false-positives and under the assumption that larger fold-change thresholds will preferentially identify the genes with the most significant biology, which would include the genes that are actually driving disease. In cases where lists of DEGs from different data sets are compared, use of a measure of agreement such as “normalized percentage of overlapping genes related” [18] can prevent the false impression of poor agreement between data sets that can result from comparing “percentage of overlapping genes” due to high sensitivity of DEG sets to small measurement variations [19]. The use of a gene-set approach, such as GSEA, may also be helpful in this regard. In psoriasis, this approach [8] has shown a larger extent of agreement between data sets than was appreciated by looking at the overlap between sequence sets.

The most interesting observation was the consistency between data sets in the direction of regulation. This consistency was obtained despite many differences between studies ranging from patient population to microarray hybridization. Biopsies were either snap-frozen [4], [5], [6] or preserved in RNAlater [3] before RNA extraction, which was performed with a variety of kits from RNeasy and mirVana to Invisorb. The major factor contributing to differences between data sets was the microarray platform, as differences between sample sets within a platform were smaller than differences between platforms. Genes clearly up-regulated in one microarray data set were almost always up-regulated in the others provided they were well-measured (p-value cut-off), even if the fold-change values were small (close to 1.0). This effect was also observed among down-regulated genes. Genes with little or no detectable fold-change in one data set generally showed consistently low fold-changes across data sets, with a high likelihood of statistically not significant fold-change. Now that data from multiple data sets are available, it has become apparent that the common choice of a 2-fold change cut-off for selecting DEGs in the case of psoriasis can be lowered, with cut-off thresholds chosen based on the tolerance for misclassification and the number of sequences “desired” for subsequent experimental follow-up. As expected, tightening the p-value for classification of a gene as differentially expressed resulted in an improved ratio of agreeing vs. disagreeing genes in the classification set, whereas tighter log10(ratio) cut-offs showed limited capacity to improve the concordant vs. discordant ratio. Our analyses indicate that sets of differentially expressed genes selected with a fold-change threshold, using a minimal log10(ratio) of 0.1 (approximately 1.26 fold) will include probe sets with similar reliability. We showed that probe sets with fold-changes of low magnitude are reproducibly identified as changed and with very similar low magnitude fold-change across data sets. This smaller effect from the disease observed on the transcriptional level does not preclude a large biological effect. Such smaller effects can be amplified by additional post-transcriptional regulation or may represent a large change in a small population of cells. Genes that are consistently over-expressed at less than a two-fold change include T cell-related genes such as CD28, CD3G and TNF-α. T cells are the target of cyclosporine [20] and DAB_389_-IL2 [21], and TNF-α is the target of effective anti-cytokine biologics in psoriasis [22], thus validating the potential importance of gene expression signals observed in this range.

The large extent of agreement between any pair of studies exploring gene expression in psoriatic lesions supports the combination of multiple psoriasis data sets to enhance the strength of conclusions that can be drawn from data sets of this type. It also implies that discoveries found in single data sets should reproduce in others. A very large number of genes are affected and these genes are differentially detectable with different methodologies. Even at low fold-change values, there was high consistency between studies. Differences in clinical protocols, patient groups, and experimental platforms cause a small increase in disagreement in the direction of gene expression changes between PP and PN tissue. However, these factors do not appear to be major contributors to the extensive and clear PP versus PN differences detected consistently in our data sets and the previously published ones. Our analyses indicate that previously used thresholds can be reduced while retaining a low false-positive rate. This results in a larger list of DEGs and leaves open the opportunity to discover yet more information relevant to psoriasis biology.

## Materials and Methods

### Ethics Statement

Clinical trial NCT00867100 was conducted according to the principles expressed in the Declaration of Helsinki. All patients provided written informed consent for expression analysis of skin biopsies and for their information to be stored in the study database and used for research. Amgen protocol 20060279 was approved by the Institutional Review Boards of the Royal Adelaide Hospital, Adelaide (Australia), The Alfred Hospital, Melbourne (Australia) and Research Review Board Inc, Waterloo (Canada). The skin biopsy collection through Asterand, Detroit, MI was approved by the review board at Chesapeake Research Review Inc.

### Samples

Paired psoriatic PP and PN frozen skin punch biopsies were procured from Asterand, Detroit, MI (n = 14) and from clinical trial NCT00867100 (Amgen protocol 20060279; n = 24). The Asterand samples were from male and female patients with mild to moderate plaque psoriasis aged 23 to 71 years. These patients did not report use of medication to treat psoriasis. The patient population of NCT00867100 has been described [23]. Briefly, patients with moderate to severe psoriasis aged 19–55 years were enrolled. Patients had to have clinically stable plaque psoriasis over ≥10% of body surface area and a Psoriasis Area and Severity Index score of ≥10. Only base-line, pre-dose biopsies were analyzed in this study. The samples from these two collections are new and do not overlap with any of the existing studies.

One 5 mm (Asterand) or 6 mm (NCT00867100) punch biopsy was taken from an active psoriatic lesion and one 5 mm (Asterand) or 6 mm (NCT00867100) punch biopsy was taken from an adjacent, uninvolved area. Skin samples were immediately flash frozen in liquid nitrogen and stored at −80°C. RNA samples from GSE11903 were obtained from Dr. Jim Krueger’s laboratory (pre- and post-treatment). Raw data from previously published studies (GSE14905, 6710, 11903, and 13355 [3], [4], [5], [6]) were included in the data analysis.

### RNA Isolation

Skin biopsies were either manually disrupted with a Multi-Sample Bio Pulverizer (Research Products International, Mount Prospect, IL) and further homogenized with a TissueRuptor (Qiagen, Valencia, CA) in the presence of lysis/binding buffer or were directly homogenized with either a TissueRuptor or Polytron (Kinematica, Lucerne, Switzerland) in the presence of lysis buffer. Total RNA was isolated from frozen psoriatic PP and PN skin punch biopsies using the mirVana miRNA Isolation Kit (Applied Biosystems, Carlsbad, CA) modified to include on-column DNase treatment with RNase-free DNase (Qiagen, Valencia, CA). The integrity of the RNA samples was assessed using a Bioanalyzer 2100. Total RNA concentration was measured on an ND-1000 Spectrophotometer (NanoDrop, Wilmington, DE).

### Quantitative Reverse Transcription-PCR

Quantitative Reverse Transcription-PCR (qRT-PCR) was performed on a subset of 8 PP/PN biopsy pairs from the Asterand sample set. The house-keeping genes β-actin and UBC were used to normalize expression levels. For β-actin the primers were 5′-CCT GGC ACC CAG CAC A-3′ and 5′-GCC GAT CCA CAC GGA GTA CT-3′, and the probe was 5′-VIC-ATC AAG ATC ATT GCT CCT CCT GAG CG-3′.For UBC the primers were 5′-TGA CAA TGC AGA TCT TCG TGA AG-3′ and 5′-GGT GTC ACT GGG CTC AAC CT-3′ and the probe was 5′-VIC-TG ACT GGT AAG ACC ATC AC-3′. For the other transcripts, pre-designed Taqman® Gene Expression assays from Applied Biosystems, Carlsbad, CA were used (see Table S1). A total of 0.75 µg RNA per sample was reverse transcribed in 50 uL (final volume) reactions primed with random hexamers using the High Capacity cDNA Reverse Transcription Kit (Applied Biosystems) according to the manufacturer’s protocol. Resulting cDNA was diluted to 2 ng/µL with 1 ug/µL glycogen (Roche, Basel, Switzerland). Five micro liters (10 ng) of each cDNA preparation were amplified in duplicate 20 µL reactions using TaqMan Universal PCR Master Mix (Applied Biosystems) according to the manufacturer’s protocol. Real-time PCR was performed in 384-well optical plates on a 7900HT Sequence Detection System (Applied Biosystems) running SDS version 2.3 software with the following conditions: 50°C for 2 min and then 95°C for 10 minutes followed by 40 cycles of 95°C for 15 sec and 60°C for 1 min. Average raw cycle threshold (Ct) data were normalized to ΔCt by using the mean of both invariant genes (β-actin and UBC). Fold difference between target gene levels in PN and PP samples was calculated with the comparative Ct method using the formula 2^−ΔΔCt^.

### Microarray Analysis

Fifty nanograms of total RNA were amplified on an ArrayPlex (Beckman Coulter, Inc., Brea, CA) using the Ovation® RNA Amplification System V2 and WB reagent (Nugen, Inc., San Carlos, CA). Of the amplified cDNA, 4.4 µg was labeled using the FL Ovation™ cDNA Biotin Module V2 (Nugen, Inc.) according to the manufacturer’s recommendations. The labeled cDNA was hybridized onto Affymetrix human genome HG-U133_Plus_2 arrays (Affymetrix, Santa Clara, CA) and processed according to Affymetrix technical protocols. The average intensity of each array was scaled to a target intensity of 500.

### Published Microarray Data Sets

The following data sets were downloaded from GEO: GSE14905 [6], GSE6710 [3], GSE11903 [24], and GSE13355 [4]. Cluster analysis (Resolver 7.2; Microsoft, Redmond, WA) and principal component analysis (Partek Genomic Suite 6.5; Partek, Inc., St. Louis, MO) were used for an initial assessment of the data sets. GSE13355 was composed of arrays with clearly different average intensities. For data analysis, GSE13355 was divided into higher (n = 38, average scale factor 1.9) and lower intensity (n = 74; average scale factor 4.9) arrays, which corresponds to arrays run in 2007 versus arrays run in 2005 and 2006, respectively [4]. Two individuals each in GSE13355 (ID3690 and ID4163) and GSE14905 (ID14 and ID24) had PP/PN pairs that clustered by individual rather than PP/PN biopsy type and were excluded from the analysis. Also excluded was a scan (GSE11903, patient G, PP) that did not meet our microarray quality control criteria of minimum intensity. The data sets and number of samples included in the analysis are listed in Table 1.

### Data Analysis

The microarray CEL files from the published data sets as well as the in-house data sets were imported into Rosetta Resolver 7.2 (Microsoft, Redmond, WA). Data was preprocessed in Rosetta Resolver using their standard processing pipeline. Ratios between PP and PN groups were generated within each data set using a pipeline that consisted of: (1) the Resolver Affymetrix Rosetta Intensity Profile Builder (saves reporters), (2) the Affymetrix Rosetta Intensity Experiment Builder (group dependent), and (3) the Affymetrix Ratio Builder (no error-weighting). This pipeline calculates a weighted-average variance that includes two components, a lower bound variance based on predetermined chip-specific background noise, and a data scatter variance derived from the probe measurements within the experiment. As N increases, the data scatter variance contributes more to the final calculation. [25]. For each data set (see Table 1), values for the PP/PN log10(ratio), p-value, and average intensity for all the probe sets on the arrays were exported from Resolver (see Table S2). For fold-change comparisons with qRT-PCR, average log10(ratio) values and standard deviations were calculated for each sample set in log10 space. Additional analyses were performed using R [26], [27], [28].

To assess the extent of agreement between pairs of data sets, we examined all possible pair-wise comparisons. For each pair, we compared the log10(ratio) for each sequence between the two data sets: if the average log10(ratio) was greater than or equal to zero or less than or equal to zero in both cases, it was classified as agreeing; if the log10(ratio) did not agree in sign, it was classified as disagreeing. Subsets of probe sets based on log10(ratio) values or p-values were created and agreements computed for p-values ranging from 0.01 to 0.2 in steps of 0.01 in the source set.

## Supporting Information

Supplemental Table 1 Taqman assays and microarray sequence codes for Taqman-microarray comparisons.
(XLSX)

Supplemental Table 2 Complete list of average log (ratio) and p-values for each data set.
(XLSX)
